# Effect of Different Root Canal Drying Protocols on the Bond Strength of Two Bioceramic Sealers

**DOI:** 10.1055/s-0042-1758807

**Published:** 2022-12-19

**Authors:** Karine Santos Frasquetti, Lucila Piasecki, Alexandre Kowalczuck, Everdan Carneiro, Vânia Portela Ditzel Westphalen, Ulisses Xavier da Silva Neto

**Affiliations:** 1 Postgraduate Program in Endodontics, School of Health and Biosciences of PUCPR, Curitiba, Paraná, Brazil; 2 Department of Periodontics and Endodontics, School of Dental Medicine, University at Buffalo, Buffalo, New York, United States

**Keywords:** bioceramic sealers, endodontic sealer, bond strength, drying protocol, push-out test

## Abstract

**Objectives** This study evaluated *in vitro* the effect of two different drying protocols on the dentin bond strength of two different bioceramic sealers (Sealer Plus BC [SP] and Bio C Sealer [BCS]). Bond strength and failure mode were evaluated according to the sealer, drying protocol, and root canal third.

**Materials and Methods** Sixty extracted human mandibular single-rooted premolars were selected after anatomical standardization. The crowns were sectioned and root canals were prepared. Roots were randomly divided into four groups ( *n* = 15 each). Each group was assigned a combination of one of the evaluated sealers (SP or BCS) and one of the drying protocols: canals dried with paper points (PP) or irrigation with saline followed by aspiration with silicon cannulas (IA). Obturations were performed using a single-cone technique. The teeth were temporized and stored for 7 days (100% humidity, 37°C). Roots were cut to obtain 2 mm thick discs for each third (coronal, middle, and apical). Push-out tests were performed on a universal testing machine, and the bond strength (MPa) of each specimen was calculated by dividing the load (N) by the interface area. Failure type was assessed under ×4 magnification.

**Statistical Analysis** Data were statistically analyzed with a significance set at 5%. An analysis of variance test followed by the Games-Howell post-hoc test was used to compare the mean values between the groups and the interaction of the variables.

**Results** The predominant failure type was cohesive, followed by mixed failure and adhesive in all groups. The apical third presented the highest bond strength ( *p* < 0.05) regardless of the group, followed by the middle and coronal thirds. Overall, the SP PP group had the highest mean bond strength ( *p* < 0.01), but the SP sealer was negatively affected by the IA drying protocol in the coronal and middle thirds. The BCS presented similar results within the third stage, regardless of the drying protocol.

**Conclusions** Sealer Plus BC had a higher bond strength than Bio C Sealer, but it was negatively affected by the irrigation-aspiration protocol in the coronal and middle thirds. For the apical third, there was no difference between the groups; thus, a similar bond strength was observed regardless of the drying protocol or sealer.

## Introduction

In endodontic therapy, obturation is the final step, in which the space of the root canal system (RCS) is filled with inert or antiseptic materials to obtain a three-dimensional seal. 1 The adhesion of filling materials to the dentin wall is a crucial step because failure in three-dimensional sealing, whether apical or lateral, might allow the invasion of microorganisms, which could result in treatment failure. 2

Although there is no ideal endodontic sealer, additional biological properties are desirable and might potentially enhance the effectiveness of root canal treatment. 3 Bioceramic sealers present several favorable properties such as alkaline pH, antibacterial activity, adequate radiopacity, and biocompatibility, in addition to not undergoing volumetric contraction and being chemically stable in a biological environment. 3 4 5 6 Another advantage is their ability to form hydroxyapatite during the hardening process, which directly influences the union between dentin and filling material. 6 7

Additionally, bioceramic sealers are also convenient because most commercially available brands present the material ready to use or with self-mixing tips that can be inserted immediately into the RCS without previous manipulation. Overall, these sealers have a working time of approximately 4 hours, 8 and the complete set is only achieved when the materials are exposed to humid environments. 8 9

A previous study 10 showed that the properties of an epoxy resin sealer were different when comparing portions prepared using the material at the beginning, middle, or end of their container. 10 Therefore, it is suggested that, because bioceramic sealers are ready-to-use materials, the moisture of the material itself may be impaired, leaving only the dentin moisture for the material to harden. 4 When inserted into a dehydrated medium, the setting time of bioceramic sealers tends to increase, which can lead to weakening of their bonding properties. 3

The presence of humidity can negatively influence sealer properties and, depending on the formulation of the material, inhibit, delay, or accelerate the setting reaction, which increases the chances of infiltration, 9 11 whereas excessive drying might remove residual water and impair the penetration of hydrophilic sealers into the dentinal tubules. 12

Bio-C Sealer (BCS; Angelus, Londrina, PR, Brazil) has been recently introduced into the market, containing calcium silicate, calcium aluminate, calcium oxide, zirconium oxide, iron oxide, and silicate dioxide, but the dispersing agent has not been disclosed by the manufacturer and has a working time of approximately 60 minute and a setting time of 120 minute. Another bioceramic sealer, Sealer Plus BC (SP; MK Life, Porto Alegre, RS, Brazil), contains calcium disilicate, calcium trisilicate, zirconia, calcium hydroxide, and polyethylene glycol as a dispersing agent; its according to the manufacturer, both working and setting times are more than 4 hours. Previous studies have shown that SP and BCS are biocompatible, bioactive, and have adequate physicochemical properties but show higher solubility than ISO 6876:2012. 13 14 15 Since calcium silicate-based materials can exhibit both solubility and fluid absorption simultaneously, 13 14 it is important to evaluate different situations that might affect their behavior. To the best of our knowledge, no previous studies have reported the bond strength of these two endodontic sealers or the effect of different RCS drying protocols on their adhesion to dentinal walls.

The aim of this study was to evaluate the effects of two different drying protocols on the dentin bond strength and failure mode of two different bioceramic sealers: SP and BCS. The null hypothesis (H0) of this study was that there is no statistically significant difference between the mean values of the variable bond strength (MPa) according to the sealer, drying protocol, and root canal third.

## Materials and Methods

After obtaining approval from the local ethics committee (4.210.161.), 60 extracted human mandibular single-rooted premolars were obtained from a local tooth bank. It included only teeth with straight roots and a single canal with a root length of at least 15 mm. The teeth were evaluated under an operating microscope at 10× magnification to exclude roots presenting with cracks, calcified canals, immature apices, resorptive defects, caries, oval canals, 16 or curvatures more than 10 degrees. 17 The teeth were scanned by cone-beam computed tomography using Scanora 3D (Soredex, Tuusula, Finland) to evaluate and standardize the anatomy of selected teeth. All teeth were kept in 0.5% chloramine-T solution until use, washed thoroughly with saline solution, and then dried. The crowns were removed using carborundum discs and the root length was set to 15 mm. A manual #10 K-file (DentsplySirona, Ballaigues, Switzerland) was used to confirm the patency. The working length (WL) was defined by subtracting 1 mm from the measurement obtained when placing the #10 file up to the apical foramen under magnification.

All samples were prepared using a single-file reciprocating V File 50 instrument (#50//.05) (TDKaFiles Shenzhen Superline, Guangming, China). Irrigation was performed using 2.5% NaOCl delivered using a plastic syringe and Navitip needle (30 G, Ultradent, South Jordan, Utah, United States) calibrated at a 1 mm shot to the WL. The final irrigation was performed using 1 mL of 17% ethylenediaminetetraacetic acid, which remained inside the RCS for 1 minute, followed by 5 mL of 2.5% sodium hypochlorite.

The two tested drying protocols in this study were as follows:

Paper points (PP): Aspiration was performed in the coronal portion of the canals followed by absorbent PP size diameter of 50.05 (TDKaFiles Shenzhen Superline, Guangming, China). Papers points were used to dry the WL, with a minimum of 3 or until the last one appeared dry.

Irrigation and aspiration (IA): Irrigation with 10 mL of saline solution, followed by aspiration from the coronal and middle portions of the sample using flexible silicone cannulas (SS Plus, Maringá, Brazil) for 5 seconds.

The samples were randomly divided into four groups ( *n* = 15 each). Each group was assigned to a combination of drying protocols (PP or IA) and one of the tested sealers, either SP or BCS. The samples were filled using a single cone obturation technique with a V File #50.05 gutta-percha cone (TDKaFiles Shenzhen Superline, Guangming, China), sealed with glass ionomer cement (Ionofast, Biodinâmica, Paraná, Brazil), stored at 37°C, and immersed in distilled water for 7 days to allow complete hardening of the sealer.

### Push-Out Test

The roots were sectioned perpendicular to the long axis to obtain a 2-mm thick slice of each third (coronal, middle, and apical). The cuts were made using a precision diamond disk (Isomet 1000, Buehler, Lake Bluff, Illinois, United States). Following previous studies, the first and last apical sections of the roots (thickness, 2 mm) were discarded. 12

Each of the 45 slices was marked on the apical side by using a permanent marker. The push-out test was performed using a universal testing machine (EMIC DL200MF, São José dos Pinhais, PR, Brazil) at the speed of 0.5 mm/min until failure and a stainless-steel tip with a diameter of 0.40, 0.50, and 0.70 mm for the apical, middle, and coronal thirds, respectively. The marked apical part was placed in contact with the tip of the cylinder to ensure that loading forces were introduced from the apical to the coronal direction. 18

The bond strength (MPa) of each specimen was calculated by dividing the load (N) by the interface area. The interface area was calculated as follows:

A = *π* ( *R* + *r* ) √h ^2^ +( *R* − *r* ) ^2^ ,

where *π* is kept constant at 3.14, *h* is the slice thickness, *R* is the largest radius of the obturator material, and *r* is the smaller radius of the obturator material obtained in the coronal and apical diameters of each slice, respectively. 19

### Analysis of Failure Modes

The samples were further examined under an optical microscope (Olympus, Tokyo, Japan) at ×40 magnification to assess failure type as follows:

Adhesive: if the sealer is completely separated from the dentin (dentin surface free of material).

Cohesive: failure occurred inside the sealer (sealer completely bonded to the dentin surface).

Mixed: a combination of adhesive and cohesive failure occurred (sealer partially bonded to the dentin surface).

**Fig. 1:**
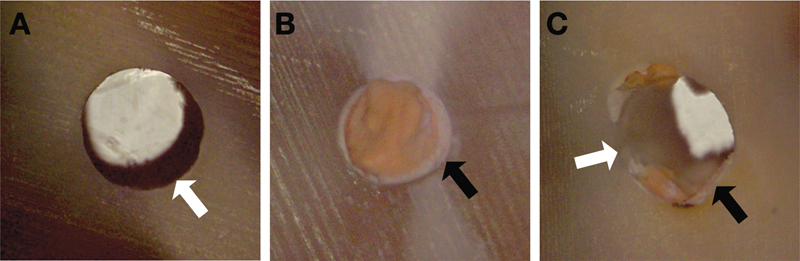
Representation of failures mode types through the optical microscope at ×40 magnification. ( **A** ) Adhesive, ( **B** ) cohesive, ( **C** ) mixed; white arrow: failures adhesives; black arrow: failures cohesives (sealer in dentin walls).

The failure mode types are represented in Fig. 1 .

### Statistical Analysis

Statistical analyses were performed using SPSS 20.0 Statistics (IBM Co., Armonk, New York, United States) with the significance set at 5%. Data were normally distributed (Kolmogorov–Smirnov test, *p >* 0.05) but not homoscedastic (Levene's test, *p <* 0.05). The power analysis showed that the sample size was adequate (99%, error rate = 0.05). An analysis of variance test followed by the Games-Howell post-hoc test was used to compare the mean values between the groups and the interaction of the variables.

## Results

**Fig. 2:**
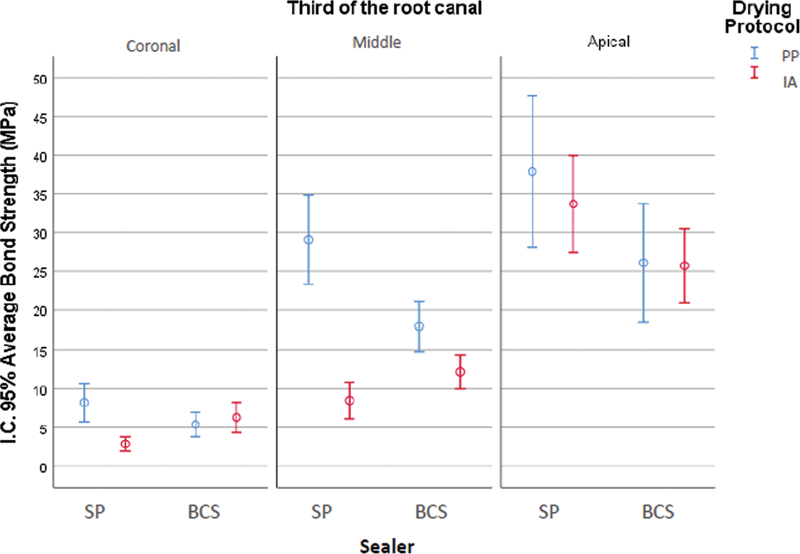
Confidence interval (95%) of average bond strength (MPa) according to third × sealer× drying protocol. BCS, Bio C Sealer; SP, Sealer Plus BC.

Table 1 presents the descriptive statistics for the variable bond strength (MPa) for each group per third and Fig. 2 shows the respective confidence intervals (95%).

**Table 1:** Descriptive statistics of bond strength (MPa) according to sealer, drying protocol, and root canal third

Group	Coronal		Middle		Apical	
	Mean	SD	Mean	SD	Mean	SD
SP PP	8.2518 aA	4.4918	29.1123 bA	10.351	37.8540 cA	17.713
SP IA	2.8984 aB	1.6839	8.5241 bB	4.2721	33.6960 cA	11.312
BCS PP	5.4053 aAB	2.8125	17.9738 bAC	5.7908	26.1158 cA	13.748
BCS IA	6.3300 aAB	3.5351	12.1688 bBC	3.9656	25.7325 cA	8.5498

Abbreviations: BC; BCS, Bio C Sealer; IA, irrigation and aspiration; PP, paper points; SD, standard deviation; SP, Sealer Plus.

Note: The lowercase letters on the line indicate the comparison between the thirds for each group. The capital letters in the column indicate the comparison within each third between the groups ( *p* < 0.05).

A statistically significant difference was observed between the thirds, regardless of the group ( *p <* 0.05), and the highest bond strength was noted at the apical level, followed by the middle and coronal thirds.

The comparison among groups showed that the SP PP group had the highest mean bond strength ( *p* < 0.01), but the IA drying protocol negatively affected the bond strength of SP sealer in the coronal and middle thirds ( *p <* 0.05). For the BCS, similar results were obtained for the tested drying protocols within each third ( *p >* 0.05). At the apical level, no statistically significant differences were observed between the groups.

**Fig. 3:**
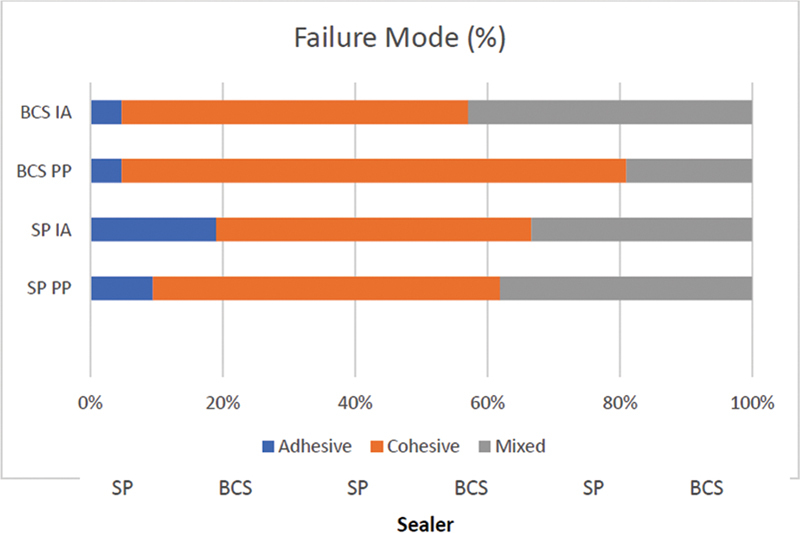
Failure mode of evaluated groups. BCS, Bio C Sealer; SP, Sealer Plus BC.

The predominant failure type was cohesive, followed by mixed failure and adhesive in all groups ( Fig. 3 ).

## Discussion

The low bond strength of the sealer to the dentin surface of RCS can affect the clinical behavior of the tooth because the material must be able to resist rupture by mechanical micro-retention, or friction must exist during dental function or root canal preparation for dental rehabilitation. 18 This study showed that the bond strengths of both brands were within the range reported in the literature for bioceramic sealers. 9 18 20 21 22

It should be noted that laboratory tests are not capable of predicting the clinical behavior of materials; since teeth are located in the alveolus, many other factors, like the periodontal ligament and the temperature of the oral cavity, can influence the properties of an endodontic sealer. However, push-out tests are useful for comparing the bond strength and evaluating the failure patterns of different materials or techniques under controlled settings, thereby minimizing bias. 22

The bioceramic sealers used in this study had different compositions, which may explain the differences observed in this study. Previous studies have shown that both SP and BCS have adequate physicochemical properties but high solubility. 11 12 13 BCS is a composite material based on calcium silicate, calcium aluminate, calcium oxide, zirconium oxide, iron oxide, and silicate dioxide, and the dispersing agent has not been described. It has a working time of approximately 60 minutes and setting time of 120 minutes after insertion into the root canal. The SP endodontic sealer contains calcium disilicate, calcium trisilicate, zirconia, calcium hydroxide, and polyethylene glycol as dispersing agents, which are different from most available bioceramic sealers. It has a working time and setting time of 4 hour. The composition and dispersing agent of a bioceramic sealer affect not only the physicochemical properties but also the biocompatibility and bioactivity of these materials. 11 These differences might explain the higher mean values of SP when used in combination with the PP drying protocol in the cervical and middle thirds.

The apical third results were significantly higher than those in the middle and coronal thirds, corroborating the results of previous studies. 9 18 These findings are likely correlated with the better adaptation of the gutta-percha cone at the apical level, which can generate higher hydraulic forces and improve the adaptation of the materials to the canal walls, resulting in a thinner sealer layer in this area. 18 Another explanation may be that bioceramic sealers contain nanoparticles that expand after the hardening reaction 23 and have hydrophilic properties. 5 24 Moreover, the small particle size (0.2 µm on average) of bioceramic sealers might result in improved distribution in the dentinal tubules, particularly in the smaller tubules of the apical third of the canals. 25 Altogether, these factors might explain the higher bond strength of the apical third, regardless of the type of sealer or the drying protocol.

The findings of this study showed that bond strength values in the coronal and middle thirds correlated with the association between the sealer and drying protocols. For SP, the use of PP resulted in significantly higher bond strength at the coronal and middle levels, whereas for BCS, similar results were found in each third, regardless of the drying protocol.

According to manufacturers, the hardening process of both sealers depends on the presence of moisture in the dentinal tubules. However, clinically, it is not possible to assess whether the amount of moisture left in dentinal tubules after the conventional use of PP could impair the hardening reaction of these materials and consequently affect their properties. Previous studies have employed a similar IA protocol to increase the moisture in the root canals during prior obturation with bioceramic sealers by irrigating the canals with solutions of isopropyl alcohol or distilled water, followed by aspiration with cannulas. 9 26 However, it is not known whether this may interfere with the properties of other sealers. Thus, in this study, the irrigation/aspiration protocol was performed with saline because it is an isotonic and sterile solution. The use of flexible silicone cannulas was chosen for this study and was expected to result in a canal that would be moist rather than completely wet or too dry. However, the overall bond strength obtained with the IA protocol was lower but significantly different only for the coronal and middle thirds when associated with SP. Previous studies have suggested that after drying the canal with PP, the remaining moisture from the dentinal tubules can maintain the hardening properties of bioceramic sealers of other commercial brands. 9 12 21 The present results corroborate these findings because the overall bond strength was higher for the PP drying protocol.

No difference was found in the failure type between the different groups and thirds evaluated in this study. The low frequency of adhesive failure suggests an adequate bond between the sealer and the dentinal walls. Notably, the present results for SP and BCS are comparable to the performance reported in the literature for other brands of bioceramic sealers. 27 28

The greater percentage of cohesive failure observed for bioceramic sealers is likely related to their physicochemical properties: different from other materials, bioceramic sealers have a continuous setting process associated with the hydration, and ionic exchange with the medium. 6 Soluble calcium hydroxide, when in contact with bioavailable phosphate, can result in the formation of hydroxyapatite. 6 7 Thus, an improvement in the long-term seal is observed owing to the formation of hard tissue where the dentin/sealer interface was initially present. 29 However, future research is needed to evaluate the bond strength of these sealers over longer periods.

## Conclusions

The bond strength was the highest in the apical third, regardless of the drying protocol or sealer. Overall, SP presented higher bond strength, but it was negatively affected by the IA drying protocol in the coronal and middle thirds, whereas BCS presented similar results within the third, regardless of the drying protocol.
